# A FRET-based high-throughput screening platform for the discovery of chemical probes targeting the scaffolding functions of human tankyrases

**DOI:** 10.1038/s41598-020-69229-y

**Published:** 2020-07-23

**Authors:** Sven T. Sowa, Carlos Vela-Rodríguez, Albert Galera-Prat, Mariana Cázares-Olivera, Renata Prunskaite-Hyyryläinen, Alexander Ignatev, Lari Lehtiö

**Affiliations:** Faculty for Biochemistry and Molecular Medicine & Biocenter Oulu, University of Oulu, Oulu, Finland

**Keywords:** Enzyme mechanisms, High-throughput screening, Assay systems, Post-translational modifications

## Abstract

Tankyrases catalyse poly-ADP-ribosylation of their binding partners and the modification serves as a signal for the subsequent proteasomal degradation of these proteins. Tankyrases thereby regulate the turnover of many proteins involved in multiple and diverse cellular processes, such as mitotic spindle formation, telomere homeostasis and Wnt/β-catenin signalling. In recent years, tankyrases have become attractive targets for the development of inhibitors as potential therapeutics against cancer and fibrosis. Further, it has become clear that tankyrases are not only enzymes, but also act as scaffolding proteins forming large cellular signalling complexes. While many potent and selective tankyrase inhibitors of the poly-ADP-ribosylation function exist, the inhibition of tankyrase scaffolding functions remains scarcely explored. In this work we present a robust, simple and cost-effective high-throughput screening platform based on FRET for the discovery of small molecule probes targeting the protein–protein interactions of tankyrases. Validatory screening with the platform led to the identification of two compounds with modest binding affinity to the tankyrase 2 ARC4 domain, demonstrating the applicability of this approach. The platform will facilitate identification of small molecules binding to tankyrase ARC or SAM domains and help to advance a structure-guided development of improved chemical probes targeting tankyrase oligomerization and substrate protein interactions.

## Introduction

**Figure 1:**
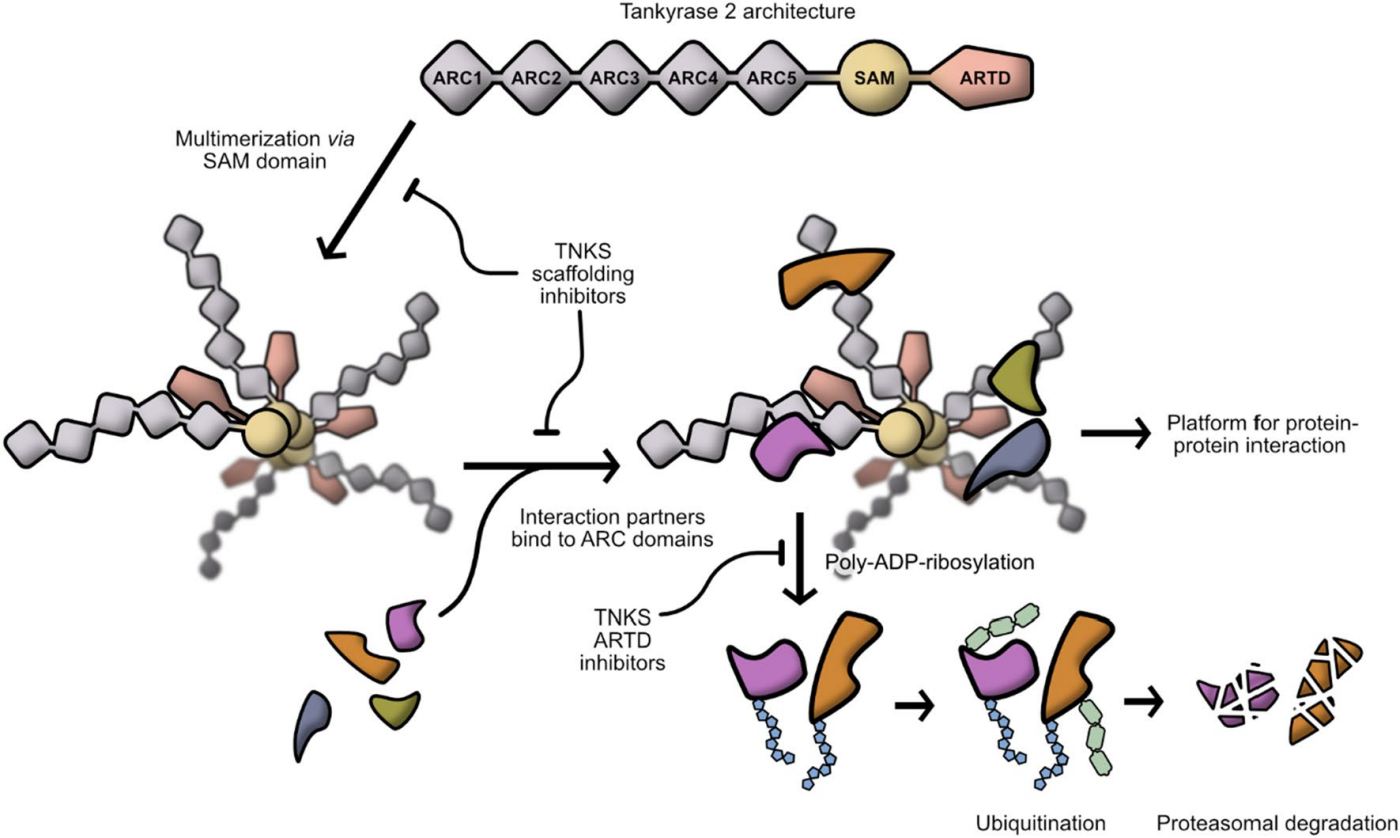
A model of TNKS function and inhibition. Tankyrases (TNKSs) are composed of five N-terminal ankyrin repeat clusters (ARCs), a central sterile α-motif domain (SAM) and the C-terminal catalytic ARTD domain. As important step of their activity as scaffold proteins, tankyrases form multimers through SAM-dependent oligomerization. Binding partners of tankyrases associate with the ARC domains. TNKS complexes might act as platforms for protein–protein interaction or lead to the poly-ADP-ribosylation of the binding partners, signalling their subsequent ubiquitination and thereby their proteasomal degradation. Inhibitors of TNKS ARTD domain prevent poly-ADP-ribosylation of binding partners and have been extensively developed and studied in recent years. An alternative approach could be to target the scaffolding function of TNKS by inhibiting either SAM-mediated multimerization or ARC-binding or protein-partners.

Poly(ADP-ribosyl) polymerases (PARPs) called tankyrases (TNKSs) are key regulators of diverse cellular processes such as mitotic spindle formation, telomere homeostasis, Wnt/β-catenin signalling and glucose metabolism^1–5^. In humans, two tankyrases with overlapping functions exist and are termed TNKS1 and TNKS2^6,7^. Like other enzymes of the PARP family, tankyrases catalyse the transfer of multiple ADP-ribose units to their protein substrates^8–10^, thus leaving them poly-ADP-ribosylated. In many cases this serves as a signal for subsequent ubiquitination and thereby proteasomal degradation^11,12^. Prominent targets of tankyrases include Axin1/2^13,14^, a major regulator of β-catenin levels, TRF1^7,9,15,16^, a telomere binding protein that inhibits telomere extension and NuMA^17–19^, a protein involved in the formation of spindle-poles during mitosis. The major role of tankyrases in the regulation of β-catenin levels has led to the development of multiple TNKS inhibitors^2,20–22^. These inhibitors function by binding to the NAD^+^ binding pocket of the catalytic ARTD domain (ADP-ribosyl-transferases diphtheria toxin-like), inhibiting the poly-ADP-ribosylation function. However, it has been shown that tankyrases also contribute through oligomerization and mediation of protein–protein interactions to the Wnt-signalling pathway^23^. We refer to these non-catalytic functions of tankyrases as scaffolding functions. By being able to specifically target different domains involved in the scaffolding function with a tool compound, it could be possible to investigate the contribution of the scaffolding to different pathways, cell and tissue types or stages of the cell cycle. A schematic representation of a current model of tankyrases function and inhibition is shown in Fig. 1.

A characteristic feature of tankyrases is the formation of complexes through its sterile α-motif (SAM) domain. This multimerization has been found to activate its catalytic function and is crucial for organization of tankyrases into cellular signalling complexes, in which they act as scaffolding proteins^23–25^. The SAM domain is a small 10 kDa α-helical fold and is found in many proteins as a motif responsible for protein–protein interaction and is often involved in oligomerization processes^26–28^. In tankyrases, the SAM domains form helical multimers by head-to-tail interaction of their end helix (EH) and mid loop (ML) surfaces^23–25^. The molecular mechanism by which SAM domain-mediated multimerization leads to increased catalytic activity of tankyrases is not fully understood. One possible mechanism was suggested in which the ARTD domains of tankyrases are brought into proximity upon multimerization and form dimers, leading to a conformational change increasing the catalytic activity^29^.

The ankyrin repeat cluster (ARC) domains of tankyrases are responsible for the binding of their interaction partners which contain degenerate RXX(P/G)XG or RXXX(P/G)XG sequence motifs^16,30–32^. Variations of these motifs determine binding affinity to tankyrases, and often multiple TNKS binding motifs (TBMs) are found in the sequence of one interaction partner^33^. Further, despite having high sequence similarity, the five ARC domains show different binding behaviour to various TBMs. ARC3 shows the least conserved sequence and studies have shown that it is not involved in binding of known TBMs and might provide a structural role^16,31^. In a proof-of-concept study, macrocyclized peptides binding to TNKS ARCs were developed^34^ based on an optimized TBM sequence^31^. These cell-permeable peptides were shown to compete with Axin for the binding of tankyrases and thus led to suppression of Wnt-signalling in cells. More recently, small molecules binding to TNKS ARC domains have been discovered by either virtual screening^35^ or fragment-based screening using differential scanning fluorimetry combined with an NMR-based approach^36^.

Taken together, the functions of tankyrases are highly dependent on their SAM domain mediated multimerization and binding of proteins by the N-terminal ARC domains. Inhibition of these scaffolding functions by small molecules could lead to the development of a novel class of highly potent TNKS inhibitors with little off-target effects and distinct biological activities. Additionally, such compounds would have a tremendous benefit as chemical probes for investigating the scaffolding function of tankyrases in the cell. So far, inhibitors of SAM domain interactions have not been reported. While there are recent reports on the early development of small chemical molecules targeting TNKS ARCs^34–36^, a suitable assay system for high-throughput screening is still missing. In our work, we developed in vitro assay systems based on fluorescence resonance energy transfer (FRET) to screen for inhibitors of TNKS scaffolding functions. This assay is based on the interaction of individual TNKS ARC domains with a TBM peptide and the interaction of dimer forming TNKS SAM mutants. The proteins were expressed as fusions with fluorescent proteins mCerulean (CFP) or mCitrine (YFP), producing a FRET signal upon interaction. Disruption of these protein pairs by small molecules results in loss of the FRET signal. We present here a robust, fast and cost-effective high-throughput assay to screen for inhibitors of tankyrase multimerization and protein partner binding function. Through validatory screening, we identified two compounds binding to the TNKS2 ARC4 domain, demonstrating the capability of the assay in identifying small molecules binding to TNKS ARCs.

## Results

### Assay setup and principle

**Figure 2:**
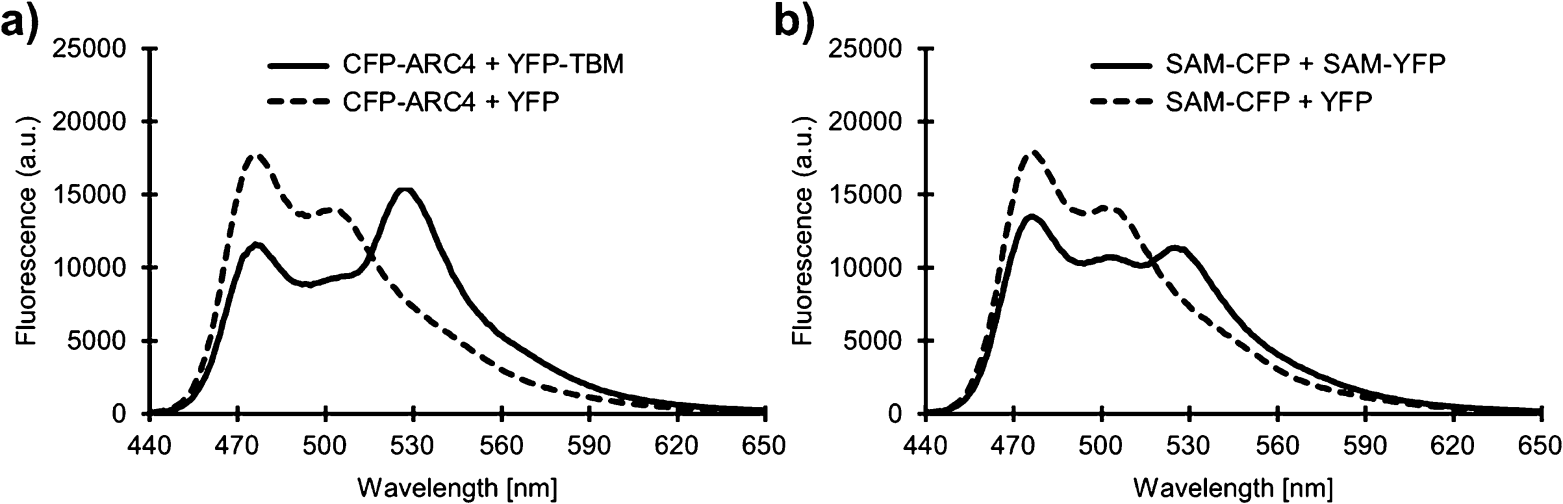
Fluorescence spectra of the TNKS2 ARC4-TBM and TNKS2 SAM FRET pairs. (**a**) Fluorescence spectra of 1 µM CFP-ARC4 (TNKS2) fusion protein mixed with 1 µM of YFP-TBM (solid line) or YFP (dashed line). (**b**) Fluorescence spectra of 1 µM TNKS2 SAM(E897K)-CFP fusion protein mixed with 1 µM TNKS2 SAM(Y920A)-YFP fusion protein (solid line) or YFP (dashed line).

Constructs of all individual ARC domains from TNKS1 and TNKS2 were recombinantly expressed as fusion proteins with CFP and subjected to purification procedures. In addition to the single ARC domains, ARC2-3 fusion constructs were produced so as not to disrupt the interconnecting α-helix, which might lead to folding issues. From all constructs expressed, CFP fusions of TNKS2 ARC2, ARC2-3 and ARC3 were insoluble and could not be purified. Information about all constructs expressed for this study is shown in Table S1. As interaction partner the previously described 8-mer peptide containing the TBM with the optimized sequence REAGDGEE^31^ was expressed at the C-terminus of YFP. Similarly, the dimer forming mutants of the TNKS1 and TNKS2 SAM domains were expressed as fusion proteins with CFP and YFP. In this study, we expressed the ML surface mutants TNKS1 SAM(E1050K) and TNKS2 SAM(E897K) fused to the N-terminus of CFP and the EH surface mutants TNKS1 SAM(Y1073A) and TNKS2 SAM(Y920A) fused to the N-terminus of YFP. The structural basis for the SAM-SAM interaction and choice of mutants is shown in Figure S1. The fluorescence spectra for CFP-fused TNKS2 ARC4 when mixed with YFP-TBM and TNKS2 SAM(E897K) mixed with TNKS2 SAM(Y920A)-YFP are shown in Fig. 2. After mixing of the FRET pairs, the ratiometric FRET signal (rFRET) is determined by calculating the ratio of the fluorescence intensity of the YFP emission wavelength (527 nm) to the fluorescence intensity of the CFP emission wavelength (477 nm) upon excitation of CFP at 410 nm. Upon interaction resulting in FRET the CFP emission decreases and the YFP emission increases thus increasing the rFRET signal. By using YFP without a fused TBM or SAM domain as control, the loss of the ratiometric FRET signal could be demonstrated.

### Comparison of FRET constructs

To compare the FRET signals of the expressed constructs, we measured rFRET of the ARC and SAM constructs by mixing 250 nM of CFP-fused proteins with 500 nM of their respective YFP-fused binding partners and estimated their binding affinities.

While it is not possible to directly infer the binding affinities from ratiometric measurements, we adapted the method described by Song et al.^37^ to determine the dissociation constants from FRET measurements for our constructs. The FRET emissions were calculated by mixing FRET donors (CFP-fusions) with increasing concentrations of FRET acceptors (YFP-fusions). We used this approach to estimate the binding affinities for all purified FRET constructs used in this study (Table 1, Figures S2 and S3). While this is an indirect approach and assumes complete maturation rate of the fluorophores and correctly determined concentrations of the proteins, the results will serve as an estimation and relative comparison of the binding affinities of the expressed FRET constructs.

**Table 1:** Dissociation constants of TNKS1/2 ARC and SAM FRET pairs.

Constructs		Dissociation constant, K_d_ [nM] (profile likelihood 95% CI)	
FRET donor	FRET acceptor		
(CFP-fusion)	(YFP-fusion)		
TNKS1 ARC1	TBM (REAGDGEE)	2,600	(2,200–3,100)
TNKS1 ARC2	TBM (REAGDGEE)	8.2	(3.7–14)
TNKS1 ARC2-3	TBM (REAGDGEE)	12	(5.3–22)
TNKS1 ARC3	TBM (REAGDGEE)	n.d	
TNKS1 ARC4	TBM (REAGDGEE)	45	(32–60)
TNKS1 ARC5	TBM (REAGDGEE)	27	(17–40)
TNKS2 ARC1	TBM (REAGDGEE)	970	(890–1,100)
TNKS2 ARC4	TBM (REAGDGEE)	35	(27–45)
TNKS2 ARC5	TBM (REAGDGEE)	33	(23–45)
TNKS1 SAM(E1050K)	TNKS1 SAM(Y1073A)	560	(520–600)
TNKS1 SAM(E1050K)	TNKS2 SAM(Y920A)	300	(270–330)
TNKS2 SAM(E897K)	TNKS1 SAM(Y1073A)	300	(270–330)
TNKS2 SAM(E897K)	TNKS2 SAM(Y920A)	230	(210–250)

The dissociation constants determined for all purified CFP-ARC constructs with YFP-TBM and constructs containing CFP- and YFP-labelled SAM domains. The profile likelihood 95% confidence interval is shown in brackets. The graphs for binding affinity determination of all constructs are shown in Figures S2 and S3. The dissociation constant for ARC3 was not determined (n. d.)

**Figure 3:**
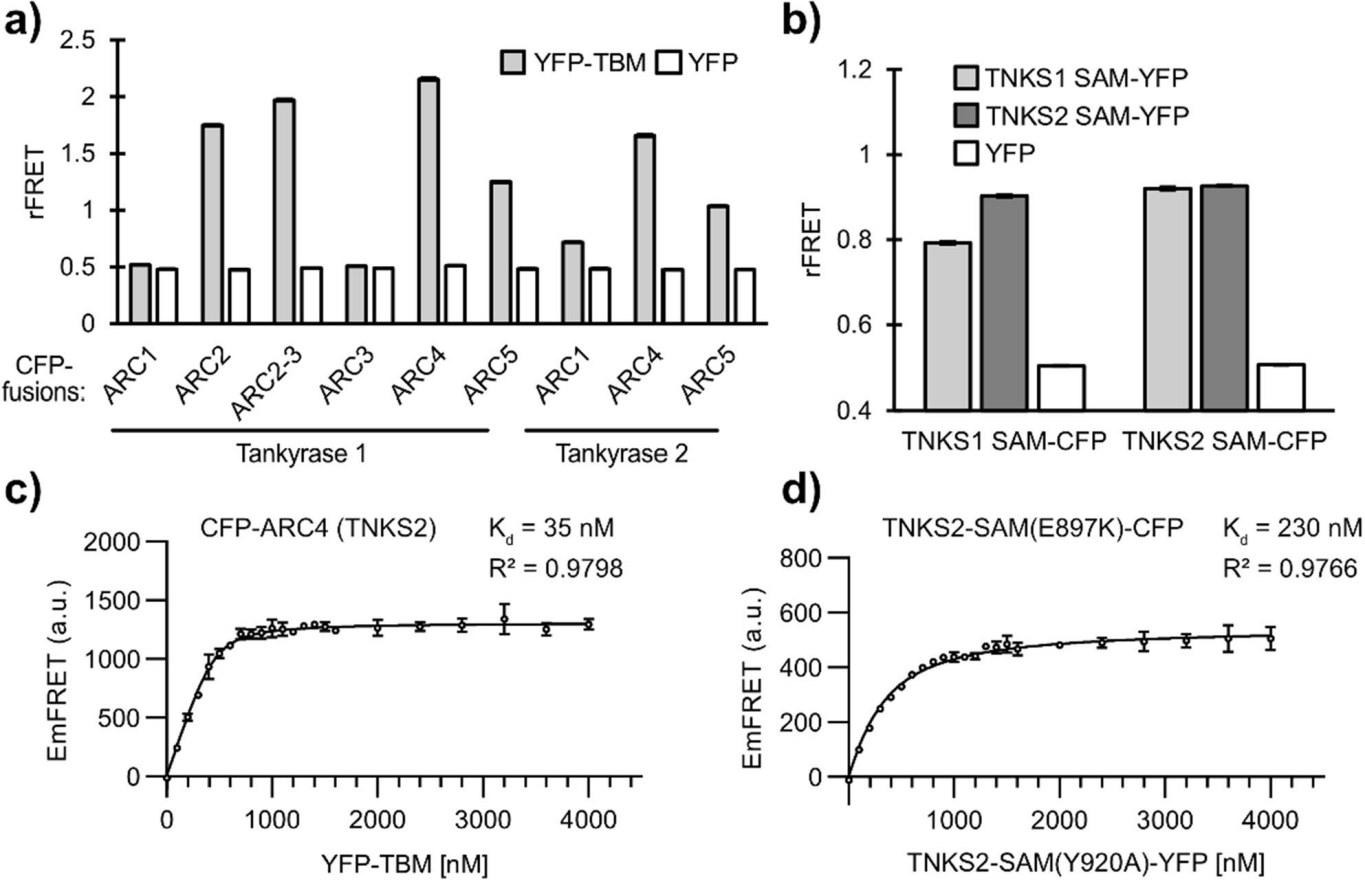
Comparison of the FRET signal from the expressed TNKS ARC and SAM constructs. (**a**) 250 nM of CFP-labelled TNKS1 and TNKS2 ARC domain constructs were mixed with 500 nM of YFP-TBM or YFP, respectively. The ratiometric FRET signal (rFRET) was measured as a 527 nm/477 nm emission ratio upon excitation at 410 nm. (**b**) 250 nM of CFP-labelled TNKS1 SAM(E1050K) or TNKS2 SAM(E897K) were mixed with 500 nM of YFP-labelled TNKS1 SAM(Y1073A), TNKS2 SAM(Y920A) or YFP, respectively. (**c**) FRET-based determination of K_d_ for TNKS2 ARC4 with YFP-TBM. (**d**) FRET-based determination of K_d_ for TNKS2 SAM(E897K)-CFP with TNKS2-SAM(Y920A)-YFP. The FRET fluorescence emissions (EmFRET) were determined as described by Song et al.^37^. The graphs for binding affinity determination of all FRET constructs are shown in Figures S2 and S3. Data shown are mean ± standard deviation with number of replicates n = 4.

From ARCs-TBM interactions, ARC4 of TNKS1 showed the highest rFRET signal when comparing all TNKS1 and TNKS2 ARC constructs (Fig. 3a). Dissociation constants were determined to be 45 nM and 35 nM for TNKS1 and TNKS2 ARC4, respectively. The determined binding affinities for TNKS1 and TNKS2 ARC5 were slightly higher (K_d_ = 27 nM and 33 nM, respectively) when compared to ARC4 constructs, however rFRET signals were lower. The ARC2 and ARC2-3 constructs of TNKS1 showed similarly high signals and dissociation constants were determined to be 8.2 nM and 13 nM, respectively. The binding occurs likely only at the ARC2 domain, as ARC3 was reported to be not binding^16^. Consistent with this, the TNKS1 ARC3 rFRET signal is nearly identical to the signal of the non-binding YFP control, and the dissociation constant could not be determined. With exception of ARC3, ARC1 shows the lowest signal in both TNKS1 and TNKS2. Dissociation constants for ARC1 from TNKS1 and TNKS2 were determined to be 2.6 µM and 0.97 µM, respectively. Again, this result is consistent with reports that ARC1 is the weakest TBM-binder of the ARC domains^31,33^. We would like to point out that the weak binding TNKS1/2 ARC1 constructs (Figure S2a,g) show low FRET emission signals and that the estimations of their dissociation constants are thus likely more susceptible to inaccuracies.

For the SAM constructs, we prepared all four possible FRET-pairs by mixing the SAM ML surface mutants (CFP-fusions) of TNKS1 and TNKS2 with the SAM EH surface mutants (YFP-fusions) of TNKS1 and TNKS2 (Fig. 3b). The dimers formed by TNKS1 SAM constructs showed a lower rFRET signal and a lower binding affinity (K_d_ = 0.56 µM) when compared to TNKS2 SAM dimers (Kd = 0.23 µM). Both TNKS1/2 SAM heterodimer combinations showed a similarly high rFRET signal compared to the TNKS2 SAM dimers and the dissociation constants were both determined to be 0.30 µM.

We chose to further optimize conditions for the screening assay with CFP-ARC4 from TNKS2, as it showed a high signal relative to the other ARCs and could be produced in higher yields (ca. 1,000 mg fusion protein per litre culture medium) compared to the other constructs (5–250 mg fusion protein per litre culture medium). From the SAM constructs, we selected the TNKS2 SAM FRET-pair for the optimization of assay conditions, showing the highest rFRET signal. Curves showing the calculated FRET-emissions for the selected FRET constructs used for the determination of binding affinities are shown in Fig. 3c,d and Figures S2 and S3. In the following, the combination of CFP-ARC4 (TNKS2) and YFP-TBM will be referred to as “ARC4-TBM FRET-pair”. Similarly, the CFP-fused TNKS2 SAM(E897K) and YFP-fused TNKS2 SAM(Y920A) will be referred to as “SAM FRET-pair”. Based on optimization of buffer conditions (Supplementary text, Figure S4, Table S2), all further tests were performed in the assay buffer containing 10 mM Bis–Tris-Propane pH 7.0, 3%(w/v) PEG20,000, 0.01%(v/v) Triton-X100 and 0.5 mM TCEP.

### Signal verification and optimization

**Figure 4:**
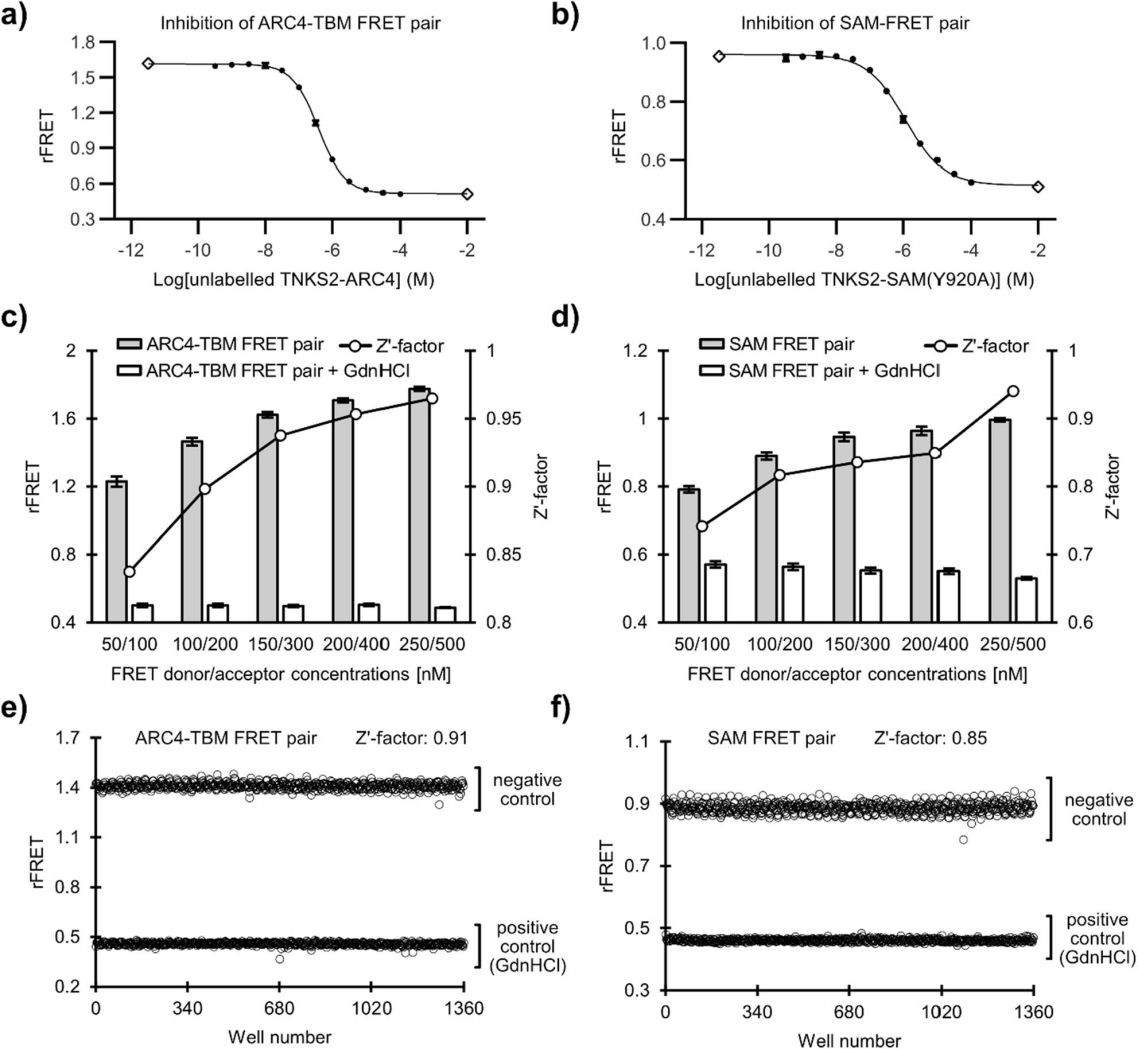
Signal quality of FRET pairs in optimized conditions. (**a**) The TNKS2 ARC4-TBM FRET pair was mixed with increasing amounts of unlabelled TNKS2 ARC4. (**b**) The TNKS2 SAM FRET pair was mixed with increasing amounts of unlabelled TNKS2 SAM(Y920A). Controls were placed 2-logarithm units above or below the highest or lowest unlabelled protein concentrations (open diamonds). Data shown are mean ± standard deviation with number of replicates n = 4. The rFRET signal of FRET pairs TNKS2 ARC4-TBM (**c**) and TNKS2 SAM (**d**) were determined at varying concentrations of the pairs, ranging from 50 nM FRET donor/100 nM FRET acceptor to 250 nM FRET donor/500 nM FRET acceptor. The FRET donor to acceptor ratio was kept constant at 1:2. Additionally, controls of the FRET pairs containing 1 M GdnHCl were measured and used as positive controls for calculation of the Zʹ-factor (solid line). Data shown are mean ± standard deviation with number of replicates n = 48. (**e**) The Zʹ-factors were determined for TNKS2 ARC4-TBM FRET pair (100 nM FRET donor/200 nM FRET acceptor) and (**f**) TNKS2 SAM FRET pair (150 nM FRET donor/300 nM FRET acceptor). As positive control, the FRET pair contained 1 M GdnHCl, resulting in a loss of the FRET signal, corresponding to 100% inhibition of the respective assay signal. For each positive and negative control, 680 replicates were dispensed in a 1536-well plate (6 µl per well) and rFRET was measured.

To confirm that competing molecules would result in a loss of the FRET signal in the assay, unlabelled TNKS2 ARC4 or TNKS2 SAM(Y920A) proteins were mixed with the respective FRET pairs (Fig. 4a,b). In both cases, the increase of unlabelled protein concentration resulted in lower rFRET signal, indicating that unlabelled proteins are able to compete with their CFP- and YFP-fusion versions. We expect that small molecules inhibiting the binding of the FRET pairs would show similar behaviour as the competing unlabelled proteins used in these experiments.

We further tested the signal of both selected FRET pairs at varying concentrations, keeping a constant FRET donor (CFP) to FRET acceptor (YFP) ratio of 1:2 (Fig. 4c,d). As a control, we included the respective FRET pairs containing 1 M guanidine hydrochloride (GdnHCl) as chaotropic agent, resulting in the loss of the FRET signal by disruption of the protein–protein interactions. This method for the determination of the Zʹ-factor was adapted from Song and Liao^38^ and addition of 1 M GdnHCl showed little to no effect on the fluorescence of CFP or YFP (Figure S5). Each datapoint was prepared in 48 replicates. With both FRET pairs, the Zʹ-factor was above 0.7 starting at the lowest tested concentration, while signal quality with a Zʹ-factor above 0.5 is considered to be excellent^39^. The ARC4-TBM FRET pair shows consistently higher Zʹ-factors at the respective concentrations compared to the SAM FRET pair, likely due to its higher rFRET signal originating from either the higher binding affinity or more optimal fluorophore orientation. We decided to use 100 nM CFP-fused TNKS2 ARC4 and 200 nM YFP-fused TBM for the ARC4-TBM FRET assay as well as 150 nM CFP-fused TNKS2 SAM(E897K) and 300 nM YFP-fused TNKS2 SAM(Y920A) for the SAM FRET assay.

After determining suitable conditions for the assays, we proceeded to assess the signal performance by comparing signal variability of conditions producing a FRET signal (negative control) or resulting in a loss of the FRET signal (positive control). Here, we used 1536-well plates, in which 690 replicates of the negative control and 690 replicates of the positive control were prepared and rFRET was measured (Fig. 4e,f). The positive controls were prepared in the same conditions, however they contained 1 M GdnHCl, resulting in the disruption of the protein–protein interactions and loss of the FRET signal corresponding to full inhibition of the interactions. For the ARC4-TBM FRET pair and SAM FRET pair, the Zʹ-factors were determined to be 0.91 and 0.85, respectively. This shows that even in 1536-well plates with a low assay volume of only 6 µl the assays show high signal quality. While the assays have good performance at low volumes in high-density plates, we note that the assays work also similarly well with larger volumes in 96- and 384-well plates.

### Screening and hit validation

**Figure 5:**
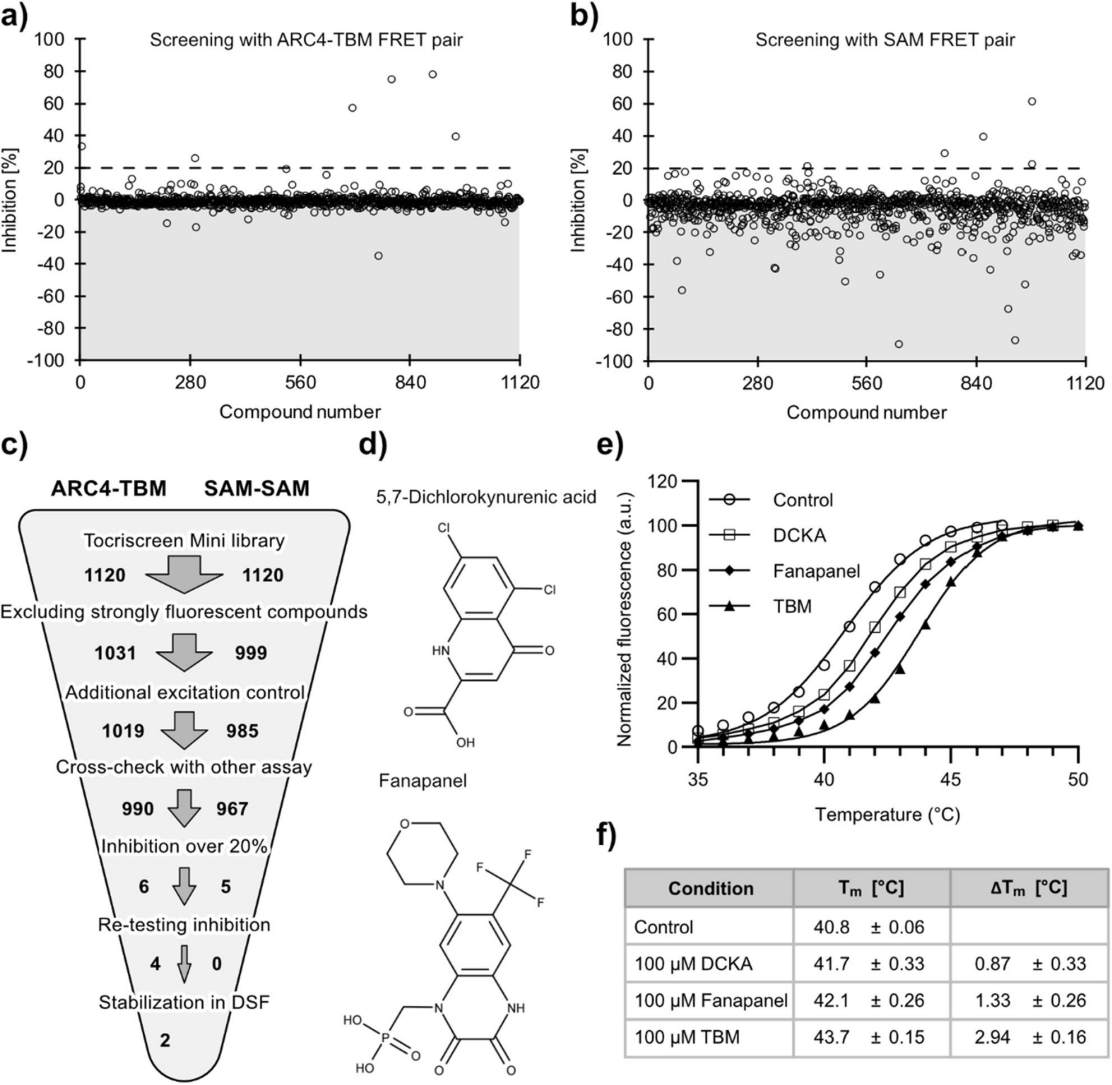
Screening of the Tocriscreen Mini library and hit validation. The percentage of inhibition was calculated relative to the rFRET signals of the positive (100% inhibition) and negative (0% inhibition) controls. A total of 1,120 compounds were screened per assay system. (**a**) Screening of the library with TNKS2 ARC4 FRET pair. In total, 130 (11.6%) measurements were excluded based on our selection criteria for interfering compounds. After removal of interfering compounds, 6 compounds (0.54%) showed over 20% inhibition (dotted line) and were regarded as hits. (**b**) Screening of the library with the TNKS2 SAM FRET pair. In total, 153 (13.7%) measurements were excluded based on our selection criteria for interfering compounds. After removal of interfering compounds, 5 compounds (0.45%) showed over 20% inhibition (dotted line) and were regarded as hits. (**c**) Scheme of the analysis for both screening procedures, with each step excluding a subset of compounds from further analysis. (**d**) Structures of the final 2 hit compounds Fanapanel and 5,7-dichlorokynurenic acid (DCKA) from the screening with ARC4-TBM. (**e**) Confirmation of hit compounds binding to TNKS2 ARC4 by DSF analysis. Melting curves of TNKS2 ARC4 were recorded with addition of 100 µM of 8-mer TBM peptide, DCKA or Fanapanel. A control containing no compound was used. (**f**) Summary of the melting temperatures (T_m_) of TNKS2 ARC4 in the presence of compounds and TBM as determined from four replicate measurements (Figure S7) and T_m_ shifts relative to the control.

We screened the Tocriscreen Mini library (Tocris Bioscience), containing 1,120 biologically active compounds to evaluate the behaviour of the assay systems under screening conditions (Fig. 5a,b). As protein–protein interactions are known to be more challenging to target than enzymes^40^ and there are only a few inhibiting molecules reported for TNKS ARCs and no inhibitors yet described for the SAM interactions, we reasoned to screen at a high compound concentration of 100 µM. At this concentration, the assays contained 1% DMSO. However, we have shown that this concentration has little effect on the assays (Figure S6).

For the screening, we combined several approaches to filter out compounds that non-specifically inhibit the FRET interaction or display intrinsic fluorescence at the wavelengths measured. The emitted fluorescence of compounds may disturb the ratiometric signal. If compounds emit fluorescence at 477 nm but none or less at 527 nm, the fluorescence intensity ratio of 527 nm/477 nm lowered and may be misinterpreted as reduction of the FRET signal.

First, we measured the fluorescence of the compounds in the assay plates before the transfer of the FRET pairs using the excitation (410 nm) and emission (477 nm and 527 nm) wavelengths of the FRET measurement. We excluded measurements that showed strong fluorescence (more than 5 times of the background fluorescence signal). Furthermore, measurements that showed fluorescence above or below 30% of the fluorescence of the respective FRET pairs compared to the controls were thought to contain compounds that interfere with the measurement and excluded from further analysis. For the ARC4 and SAM assays, 89 (7.9%) and 121 (10.8%) compounds respectively were excluded based on these criteria.

Second, we used an additional excitation wavelength (430 nm) for the FRET measurement. We reasoned that there is a high possibility for interfering fluorescent compounds to be more strongly excited at either 410 or 430 nm, resulting in a difference of the ratiometric FRET signal relative to the signal measured at the other excitation wavelength. Compounds were excluded when showing more than 10% difference in apparent inhibition using both readout settings. For the ARC4 and SAM assays, 12 (1.1%) and 14 (1.3%) compounds respectively were excluded based on these criteria.

Last, by screening the assays for ARC-TBM and SAM-SAM in parallel, any compounds resulting in signal reduction of both assays simultaneously were excluded based on the assumption that these compounds introduced non-specific disruption of the protein–protein interactions or interfered by other mechanisms such as fluorescence or quenching. The interaction of SAM-SAM and ARC-TBM is highly different. The dimerization of the SAM pair is based on the interaction of the two protein surfaces, while the ARC-TBM interaction is based on the binding of the disordered TBM to a binding pocket created by ARC domains and it is unlikely that one compound would specifically disrupt these two interactions. For the ARC4 and SAM assays, 29 (2.6%) and 18 (1.6%) compounds respectively were excluded based on these pan-assay interference criteria. In total, 130 compounds (11.6%) from the ARC4 assay and 153 compounds (13.7%) from the SAM assay were excluded (Fig. 5c). Of the excluded compounds, 114 compounds showed overlap in both assays. It is to note that we screened at a high compound concentration of 100 µM at which intrinsic fluorescence and aggregator properties are often observed. Screening at lower concentrations should result in fewer excluded measurements based on these criteria. After application of these filters, compounds that showed an inhibition of 20% or higher were considered hits. We chose this low hit limit to also include binders with low potency. For the ARC4 assay, 6 compounds (0.54%) matched the hit criteria, while for the SAM assay 5 compounds (0.46%) were considered as hits.

Re-testing of the identified hits revealed that 4 of the 6 compounds showed inhibition of ARC4-TBM, while none of the 5 initial hits for the SAM-SAM interaction showed inhibition. The 4 compounds inhibiting the TNKS2 ARC4-TBM interaction were tested for stabilization of ARC4 using differential scanning fluorimetry (DSF). Two of the compounds, 5,7-dichlorokynurenic acid (DCKA) and Fanapanel (Fig. 5d), lead to T_m_ shifts of 0.87 ± 0.33 °C and 1.33 ± 0.26 °C, respectively at 100 µM concentration, while TBM at the same concentration resulted in a T_m_ shift of 2.94 ± 0.16 °C (Fig. 5e,f). Both hits can therefore, with high likelihood, be considered binders of TNKS2 ARC4. Based on the estimation of dissociation constants for the ARC4-TBM FRET pair interaction, the K_i_ values for DCKA and Fanapanel were calculated to be 62 µM and 20 µM, respectively (Supplementary text, Figure S8). A previously described compound binding to tankyrases ARCs with similar K_i_ was demonstrated to inhibit Wnt-signalling^35^, however we could not find reduction of Wnt-signalling with DCKA or Fanapanel in a cell-based TCF/LEF reporter assay (Figure S9). Interestingly, both compounds are reported as antagonists of ionotropic glutamate receptors^41,42^ and show structural similarities such as conjugated double-ring systems and negatively charged functional groups. It is tempting to speculate that ARC4 binds both molecules in a similar manner, and that negatively charged groups might represent a structural feature beneficial for the binding of compounds to the binding pocket of ARCs, especially considering that the strongly binding TBM peptide (REAGDGEE) contains four acidic residues.

## Discussion

In recent years, tankyrases have received a lot of attention as attractive targets for the development of therapeutics against cancer and fibrotic diseases^2,20–22^. Functional studies have revealed tankyrases as regulators of many crucial cellular processes such as Wnt/β-catenin-signalling, telomere maintenance and mitosis^1–5^. While the development of TNKS inhibitors mainly focussed on the inhibition of the catalytic domain, the function of tankyrases is coupled to many regulatory processes beyond its catalytic function^3,23,25^. With newer findings about the scaffolding function of tankyrases and its contribution to the regulation of the catalytic activity, several reports have suggested that the inhibition of multimerization or protein-binding function may represent an effective way for the specific inhibition of tankyrases^23,34–36,43^. Such inhibitors would also be valuable chemical probes aiding the investigation of TNKS mechanism of function in the cell.

Here, we report the development of two assay systems based on FRET which can be used for screening of small molecules that inhibit the ARC mediated binding of protein interaction partners or SAM-based multimerization. We showed that the FRET signal originates from specific interaction of either ARC4-TBM or SAM-SAM pairs. Extensive optimization efforts have led to two assay systems with very robust signal quality (Zʹ-factor > 0.8) that can be run in 96-, 384- or 1536-well plates. The proteins required for the assays are produced recombinantly in *E. coli* in high yields and no expensive chemicals or buffer components are needed, resulting in an overall cost-effective assay setup. The Tocriscreen Mini library, containing 1,120 biologically active compounds, was used with both assay systems for validatory screening. After applying our criteria to remove interfering compounds and validation of hits, we identified 2 compounds, DCKA and Fanapanel, that inhibit ARC-TBM binding and show stabilization of the ARC4 domain in DSF. We could not observe effects of these compounds on Wnt-signalling in a cell-based TCF/LEF reporter assay, however higher compound concentrations might be needed and cellular permeability of the compounds could be limiting the effect.

In the future, the assay will help to identify small chemical inhibitors of TNKS ARC-protein binders and SAM-SAM-interactions. With only a few reported small chemical binders for TNKS ARCs to date^34–36^, we could already expand the number with our validatory screening efforts appreciably. Last, X-ray crystallographic studies of ARCs with these molecules would provide important insights into the binding modes and could lead to a structure guided design of novel chemical probes with higher affinity.

## Methods

### Cloning

Inserts of the fusion constructs were prepared by overlap-extension PCR. The expression constructs were cloned into pNIC28-Bsa4 plasmids (Addgene plasmid # 26103) using SLIC restriction free cloning method^44^. The insert encoding TNKS2 ARC4 was cloned into pNIC28-MBP. We prepared pNIC28-MBP by insertion of the sequence for *E. coli* maltose binding protein (UniProt ID: P0AEX9, MBP_27-392_) between His_6_-tag and TEV protease cleavage site of pNIC28-Bsa4. Briefly, the pNIC28-Bsa4 or pNIC28-MBP plasmids were linearized and 100 ng of linearized plasmids were mixed in a 1:3 molar ratio with PCR products of the expression constructs (Table S1) and incubated with T4 polymerase for 2.5 min at room temperature and for 10 min at 4 °C. The mixture was used to transform NEB 5α competent *E. coli* cells (New England BioLabs) according to manufacturer’s instructions. Colonies were grown on LB agar containing 5% sucrose using the SacB-based negative selection marker^45,46^. All constructs were verified by sequencing of the insert regions.

### Protein expression

Expression constructs are based on pNIC28-Bsa4 or pNIC28-MBP and contain a His_6_-tag or His_6_-MBP-tag, respectively, followed by a TEV protease cleavage site before the construct sequence (ENLYFQ*SM). The plasmid was transformed to *E. coli* BL21(DE3) cells. 500 ml Terrific Broth (TB) autoinduction media including trace elements (Formedium, Hunstanton, Norfolk, England) were supplemented with 8 g/l glycerol and 50 μg/ml kanamycin and inoculated with 5 ml of overnight preculture. The flasks were incubated shaking at 37 °C until an OD_600_ of 1 was reached. The temperature was set to 18 °C and incubation continued overnight. The cells were collected by centrifugation at 4,200×*g* for 30 min at 4 °C. The pellets were resuspended in lysis buffer (50 mM HEPES pH 7.5, 500 mM NaCl, 15 mM imidazole). For constructs containing SAM domains, a different lysis buffer was used (40 mM Bis–Tris-Propane pH 9.0, 500 mM NaCl, 15 mM imidazole). Resuspended cells were stored at − 20 °C until purification.

### Protein purification

All constructs were initially purified by immobilized metal affinity chromatography (IMAC). For some constructs, additional purification steps were performed. The cells were thawed and lysed by sonication. The lysate was centrifuged (16,000×*g*, 4 °C, 30 min), filtered and loaded onto a 5 ml HiTrap HP column equilibrated with lysis buffer and charged with Ni^2+^. The column was washed with 5 column volumes of lysis buffer and 5 column volumes of lysis buffer containing 25 mM imidazole. The protein was eluted using lysis buffer containing 300 mM imidazole. IMAC eluate of MBP-tagged ARC4 (TNKS2) protein was loaded onto a 5 ml MBPTrap HP column equilibrated with MBPTrap-loading buffer (20 mM HEPES pH 7.5, 100 mM NaCl), washed with 4 column volumes of MBPTrap-loading buffer and eluted with the same buffer containing 10 mM maltose. For the constructs ARC4 (TNKS2), CFP-ARC1 (TNKS1) and CFP-ARC5 (TNKS2), an additional treatment with TEV-protease (1:30 molar ratio, 16 h, 4 °C) to remove His_6_-MBP- or His_6_-tags was performed followed by a reverse IMAC step to remove impurities. Size exclusion chromatography was carried out on a S75 16/600 size-exclusion chromatography column with IMAC eluate of CFP-ARC4 (TNKS1) and the TEV-cleaved reverse IMAC flow through of TNKS2 ARC4 in 20 mM HEPES pH 7.5, 500 mM NaCl, 0.5 mM TCEP, 10% (v/v) glycerol or 20 mM HEPES pH 7.5, 100 mM NaCl, 0.5 mM TCEP, respectively. Proteins were aliquoted and flash frozen in liquid nitrogen and stored at − 70 °C.

### FRET measurements

Measurements were performed with the multimode microplate reader TECAN Infinite M1000 PRO. An excitation wavelength of 410 nm (20 nm bandwidth) and emission wavelengths of 477 nm (10 nm bandwidth) and 527 nm (10 nm bandwidth) were used. The number of flashes was set to 50 with a flash frequency of 400 Hz. Integration time was 20 µs and settle time was 10 ms. After subtraction of the blank for each emission measurement, the ratiometric FRET value (rFRET) was calculated by dividing the fluorescence intensity at 527 nm by the fluorescence intensity at 477 nm. For the validatory screening, the measurement was repeated with an additional excitation wavelength of 430 nm (20 nm bandwidth). Measurements were performed in either 384-well black low-volume polypropylene plates or 1536-well black polystyrene plates from Fisherbrand. With exception of buffer optimization tests, all FRET experiments were performed in assay buffer (10 mM Bis–Tris-Propane pH 7.0, 3% (w/v) PEG20,000, 0.01%(v/v) Triton-X100 and 0.5 mM TCEP).

### Determination of dissociation constants

To determine the dissociation constants of the FRET constructs, CFP-fused TNKS1/2 ARC or SAM constructs (50 nM to 1.1 µM, Figures S2 and S3) were mixed with 0, 100, 200, 300, 400, 500, 600, 700, 800, 900, 1,000, 1,100, 1,200, 1,300, 1,400, 1,500, 1,600, 2000, 2,400, 2,800, 3,200, 3,600 and 4,000 nM of YFP-TBM or TNKS1 SAM(Y1073A)-YFP or TNKS2 SAM(Y920A)-YFP, respectively. CFP-ARC3 (TNKS1) was diluted to 7.5 µM in 20 mM HEPES pH 7.5, 25 mM NaCl, 0.5 mM TCEP prior setting up the experiment, as it showed slight precipitation in the assay buffer at high concentrations. The concentrations of the respective CFP-fusion proteins were kept constant and chosen to ensure saturation of the resulting FRET-emission signals (flattening of the curves). Dispensing of proteins was done with an Echo 650 acoustic dispenser. The analysis of the data was done in GraphPad Prism 7 as described by Song et al.^37^. To calculate the FRET emission, we measured the emission at 477 nm (5 nm bandwidth) and 527 nm (5 nm bandwidth) upon excitation at 430 nm (5 nm bandwidth). Additionally, the emission at 527 nm (5 nm bandwidth) was measured upon excitation at 477 nm (5 nm bandwidth). Other parameters were kept as described for FRET measurements above. Per condition, 4 replicates were prepared in 384-well plates with a volume of 10 µl per well.

### Comparison of signals for FRET constructs

To test the rFRET signal of the different TNKS ARC and SAM constructs, 250 nM CFP-fused TNKS1-ARC1, -ARC2, -ARC2-3, -ARC3, -ARC4 or -ARC5 and TNKS2-ARC1, -ARC4 or -ARC5 were mixed with 500 nM of YFP-TBM. Similarly, 250 nM of TNKS1 SAM(E1050K)-CFP or TNKS2 SAM(E897K)-CFP protein constructs were each mixed with 500 nM of TNKS1 SAM(Y1073A)-YFP or TNKS2 SAM(Y920A)-YFP. The 250 nM of related CFP-constructs mixed with 500 nM of YFP were used as control. Per condition, 4 replicates were prepared in 384-well plates with a volume of 20 µl per well.

### Competition assay and IC_50_ measurements

CFP-TNKS2-ARC4 (100 nM) and YFP-TBM (200 nM) were mixed with increasing concentrations of unlabelled ARC4. TNKS2 SAM(E897K)-CFP (150 nM) and TNKS2 SAM(Y920A)-YFP (300 nM) were mixed with increasing concentrations of unlabelled SAM(Y920A). For measurements of IC_50_ values for the compounds 5,7-dichlorokynurenic acid and Fanapanel, CFP-TNKS2-ARC4 (50 nM) and YFP-TBM (100 nM) were mixed with increasing concentrations of compounds. Additionally, for statistical analysis, the controls without unlabelled proteins or compounds and containing 1 M GdnHCl were set 2 orders of magnitudes from the lowest and highest concentrations of mixed protein or compound dilution series, respectively. The analysis of the data was done in GraphPad Prism 7 using a nonlinear regression analysis (sigmoidal dose–response fitting with variable slope). Per condition, 4 replicates were prepared in 384-well plates with a volume of 20 µl per well.

### Assay signal verification

Experiments were done in 1536-well plates and solutions were transferred using Mantis liquid dispenser (Formulatrix). To evaluate concentrations of the constructs used for screening, the FRET signal of TNKS2 CFP-TNKS2-ARC4 and YFP-TBM as well as SAM(E897K)-CFP and TNKS2 SAM(Y920A)-YFP at increasing concentrations of the fret pairs were tested. The ratio of FRET donor to acceptor was kept constant at 1:2. The FRET donor/acceptor concentrations of 50/100, 100/200, 150/300, 200/400 and 250/500 nM were tested, and for each condition 48 datapoints were collected. Volumes were 5 µl per well.

For the calculation of the Zʹ-factors^39^, CFP-TNKS2-ARC4 (100 nM) and YFP-TBM (200 nM) or TNKS2 SAM(E897K)-CFP (150 nM) and TNKS2 SAM(Y920A)-YFP (300 nM) were mixed and rFRET signal was measured in the presence and absence of 1 M GdnHCl with 680 replicates for each condition. Volumes were 6 µl per well.

### Validatory screening

For the screening, 60 nl of compounds from the Tocriscreen Mini library (Tocris Bioscience) were transferred to 1536-well plates. The mixtures of CFP-TNKS2-ARC4 (100 nM) and YFP-TBM (200 nM) or TNKS2 SAM(E897K)-CFP (150 nM) and TNKS2 SAM(Y920A)-YFP (300 nM) were prepared in assay buffer (10 mM Bis–Tris-Propane pH 7.0, 3%(w/v) PEG20,000, 0.01%(v/v) Triton-X100 and 0.5 mM TCEP), and rFRET signal was measured at two excitation wavelengths 410 nm and 430 nm. Protein mixtures were transferred to the plates using Mantis liquid dispenser (Formulatrix).

### Differential scanning fluorimetry

The experiments were performed in assay buffer (10 mM Bis–Tris-Propane pH 7.0, 3%(w/v) PEG20,000, 0.01%(v/v) Triton-X100 and 0.5 mM TCEP) in 96-well qPCR plates. Unlabelled TNKS2-ARC4 was mixed with SYPRO Orange dye (ThermoFisher Scientific) to final concentrations of 5 µM ARC4 and 5 × SYPRO Orange and a volume of 20 µl. To determine melting temperatures, 100 µM of 5,7-dichlorokynurenic acid, Fanapanel or TBM-peptide (REAGDGEE, JPT Peptide Technologies) were added to the mixture. Additionally, controls containing no compounds were prepared. All conditions contained 1%(v/v) DMSO. Data points for melting curves were recorded in 1 min intervals from 20–95 °C, with the temperature increasing by 1 °C/min. The analysis of the data was done in GraphPad Prism 7 using a nonlinear regression analysis (Boltzmann sigmoid equation) of normalized data. Per condition, 4 replicates were prepared with a volume of 20 µl per well.

### Wnt-signalling reporter assay

The TCF/LEF reporter-HEK293 cell line (rHEK293) containing the stably integrated Wnt reporter assay was purchased from BPS Bioscience, catalog #60501. Assay was essentially performed as previously described^47^ with slight modification. Shortly, 1 mM Na-pyruvate (Gibco) and 10% foetal bovine serum containing DMEM media was used instead of serum-free media. Compounds (DCKA, Fanapanel, XAV939) were directly transferred to assay plates with an Echo 650 acoustic dispenser. Cells were incubated with compounds for 20 h before addition of control media and Wnt3a-conditioned media. All conditions contained 0.5% (v/v) DMSO.

## Supplementary information

Supplementary Information 1.
