# Supplementary material for: A FRET-based high-throughput screening platform for the discovery of chemical probes targeting the scaffolding functions of human tankyrases
Source: Sci Rep. 2020 Jul 23;10:12357. doi: 10.1038/s41598-020-69229-y (PMC7378079; doi:10.1038/s41598-020-69229-y)
Supplement: Supplementary file 1 — Supplementary Information 1. [file 41598_2020_69229_MOESM1_ESM.pdf]

Supplementary information

A FRET-based high-throughput screening platform for the discovery of chemical probes targeting the scaffolding functions of human tankyrases

Sven T. Sowa, Carlos Vela-Rodríguez, Albert Galera-Prat, Mariana Cázares-Olivera, Renata Prunskaite-Hyyryläinen, Alexander Ignatev & Lari Lehtiö*

Faculty for Biochemistry and Molecular Medicine & Biocenter Oulu, University of Oulu, Oulu, Finland

*Corresponding author: lari.lehtio@oulu.fi

Content

Figure S1: Interaction of tankyrase SAM domains and sites of introduced dimerizing mutants.

Table S1: Expression constructs.

Figure S2: Dissociation constants determined from FRET emission for expressed CFP-ARC constructs with YFP-TBM.

Figure S3: Dissociation constants determined from FRET emission for expressed SAM-CFP and SAM-YFP constructs.

Buffer optimization protocol and results.

Figure S4: Buffer pH and buffering agent optimization.

Table S2: Buffer additive optimization.

Effect of GdnHCl on CFP and YFP fluorescence.

Figure S5: Effect of GdnHCl on CFP and YFP fluorescence.

Testing of DMSO effect on assay protocol and results.

Figure S6: Effect of different DMSO concentrations on the assay systems.

Figure S7: Four replicate measurements with differential scanning fluorimetry for hit compounds of TNKS2 ARC4.

Estimation of binding affinities of hit compounds to ARC4.

Figure S8: IC_{50} measurements of two hit compounds with ARC4-TBM FRET pair.

Figure S9: TCF/LEF Wnt-reporter assay.

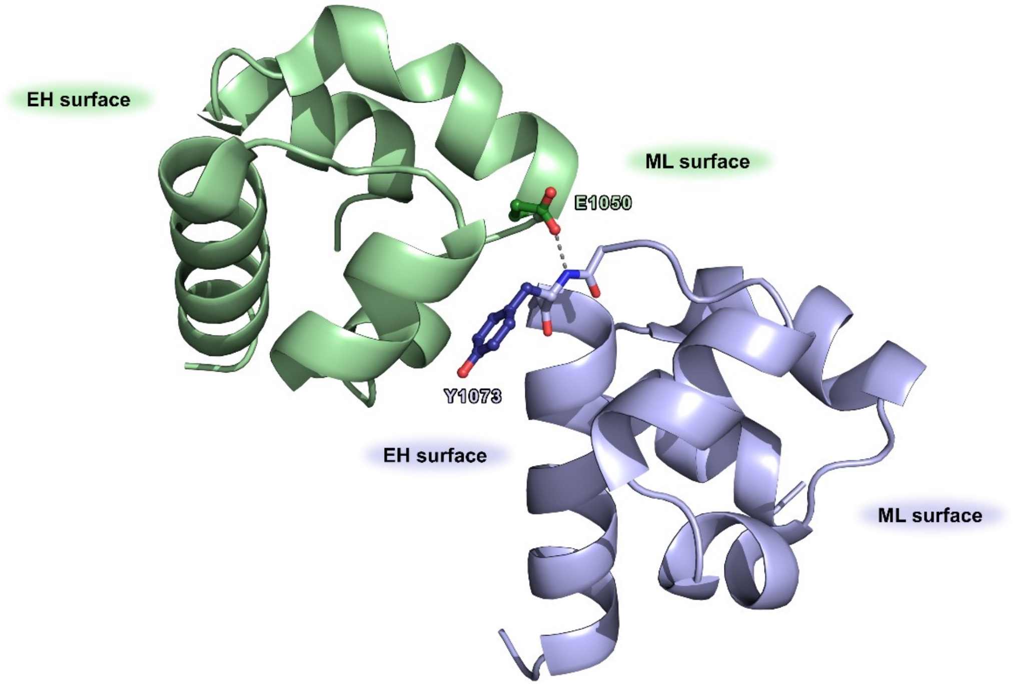

Figure S1: Interaction of tankyrase SAM domains and sites of introduced dimerizing mutants. The structure of the wildtype SAM domains of TNKS1 is shown (PDB accession code: 5KNI). The ML surface mutations used in this study are E1050K for TNKS1 and corresponding E897K for TNKS2. The glutamate residue 1050 of TNKS1 is located at the ML surface of the SAM domain and forms a hydrogen bond to a backbone amide group of the interacting SAM domain (dashed line). The EH surface mutants used in this study are Y1073A for TNKS1 and corresponding Y920A for TNKS2. Tyrosine 1073 of TNKS1 is located at the EH surface and packs against a hydrophobic patch of the ML surface of the interacting SAM domain.

Table S1: Expression constructs. The construct boundaries are based on residue numbering from the UniProt database (identifiers: TNKS1 = O95271, TNKS2 = Q9H2K2). The full sequences of the fluorescent proteins can be found from Addgene (identifiers: mCerulean = 29726, mCitrine = 29724).

Construct	Construct boundaries	Comment
CFP	mCerulean	Addgene: 29726
YFP	mCitrine	Addgene: 29724
TNKS1 SAM(E1050K)-CFP	TNKS1(E1050K)$_{1017-1086}$-GGSGGGS-mCerulean	
TNKS1 SAM(Y1073A)-YFP	TNKS1(Y1073A)$_{1017-1086}$-GGSGGGS-mCitrine	
TNKS2 SAM(E897K)-CFP	TNKS2(E897K)$_{873-936}$-SGGGS-mCerulean	
TNKS2 SAM(Y920A)-YFP	TNKS2(Y920A)$_{873-936}$-SGGGS-mCitrine	
CFP-ARC1 (TNKS1)	mCerulean-SG-TNKS1$_{178-330}$	
CFP-ARC2 (TNKS1)	mCerulean-SG-TNKS1$_{331-501}$	
CFP-ARC2-3 (TNKS1)	mCerulean-SG-TNKS1$_{331-648}$	
CFP-ARC3 (TNKS1)	mCerulean-SG-TNKS1$_{476-648}$	Precipitates over 5 µM in assay buffer containing PEG20,000.
CFP-ARC4 (TNKS1)	mCerulean-SG-TNKS1$_{646-803}$	
CFP-ARC5 (TNKS1)	mCerulean-SG-TNKS1$_{799-961}$	
CFP-ARC1 (TNKS2)	mCerulean-SG-TNKS2$_{20-175}$	
CFP-ARC2 (TNKS2)	mCerulean-SG-TNKS2$_{173-343}$	Insoluble
CFP-ARC2-3 (TNKS2)	mCerulean-SG-TNKS2$_{173-490}$	Insoluble
CFP-ARC3 (TNKS2)	mCerulean-SG-TNKS2$_{320-490}$	Insoluble
CFP-ARC4 (TNKS2)	mCerulean-SG-TNKS2$_{488-645}$	
CFP-ARC5 (TNKS2)	mCerulean-SG-TNKS2$_{641-800}$	
YFP-TBM	mCitrine-REAGDGEE	
TNKS2 ARC4	TNKS2$_{487-649}$	
TNKS2 SAM(Y920A)	TNKS2(Y920A)$_{873-936}$	

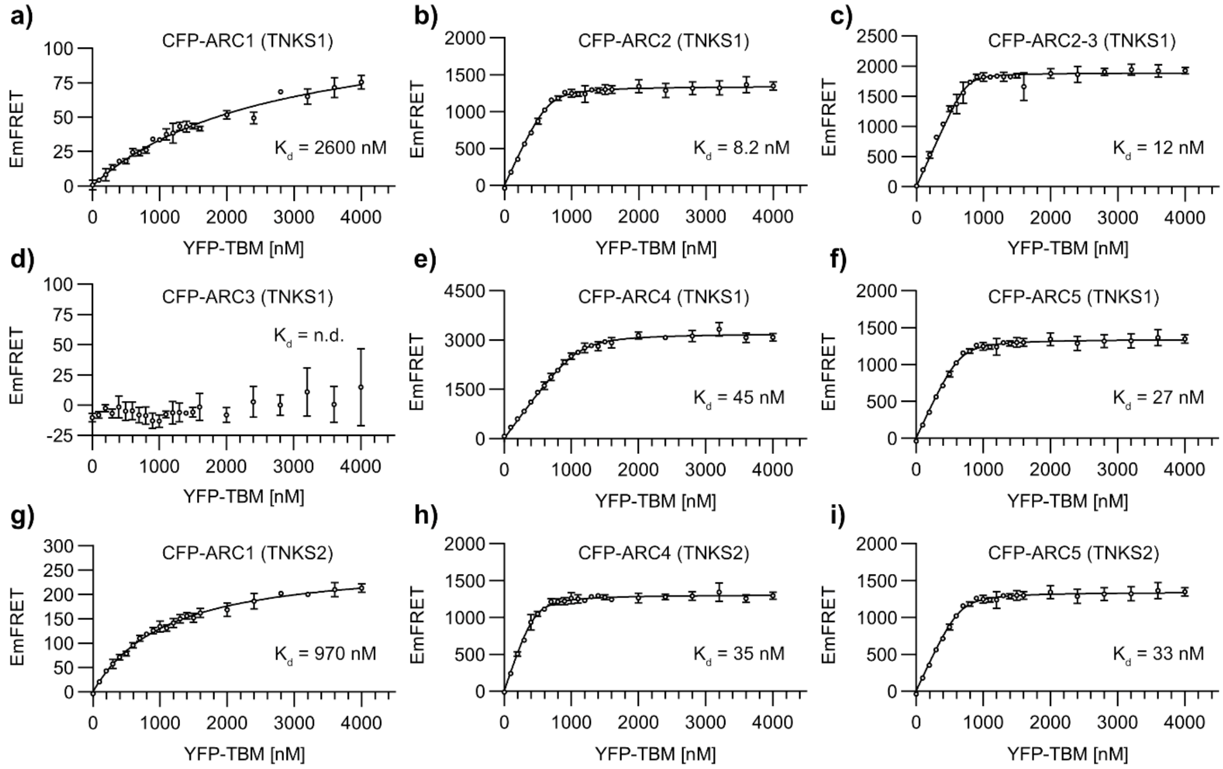

Figure S2: Dissociation constants determined from FRET emission for expressed CFP-ARC constructs with YFP-TBM. The concentration of the CFP-ARC constructs was kept constant and mixed with increasing concentrations of YFP-TBM and FRET fluorescence emissions (EmFRET) were determined. **(a)** CFP-ARC1 (TNKS1) concentration was kept at 50 nM. The determined K_d is 2,600 nM. **(b)** CFP-ARC2 (TNKS1) concentration was kept at 600 nM. The determined K_d is 8.2 nM. **(c)** CFP-ARC2-3 (TNKS1) concentration was kept at 750 nM. The determined K_d is 12 nM. **(d)** CFP-ARC3 (TNKS1) concentration was kept at 150 nM. The K_d was not determined (n.d.). **(e)** CFP-ARC4 (TNKS1) concentration was kept at 1.1 µM. The determined K_d is 45 nM. **(f)** CFP-ARC5 (TNKS1) concentration was kept at 750 nM. The determined K_d is 27 nM. **(g)** CFP-ARC1 (TNKS2) concentration was kept at 150 nM. The determined K_d is 970 nM. **(h)** CFP-ARC4 (TNKS2) concentration was kept at 450 nM. The determined K_d is 35 nM. **(i)** CFP-ARC5 (TNKS2) concentration was kept at 650 nM. The determined K_d is 33 nM. Data shown are mean ± standard deviation with number of replicates n=4.

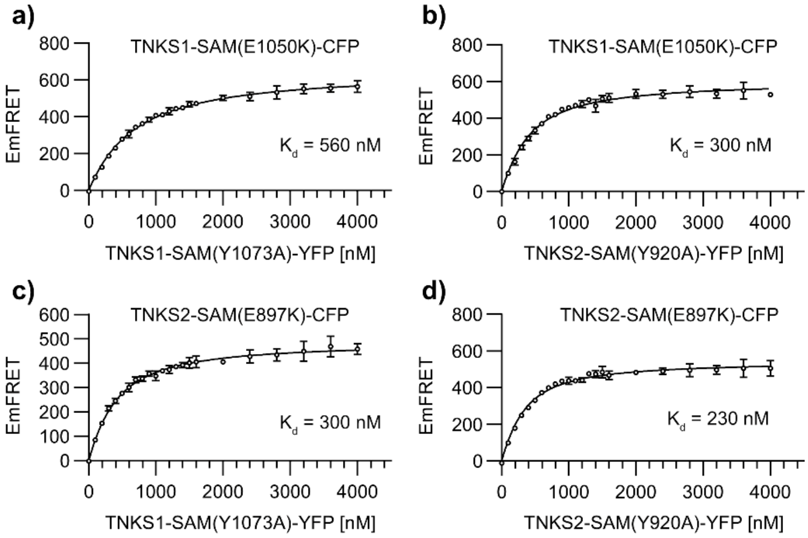

Figure S3: Dissociation constants determined from FRET emission for expressed SAM-CFP and SAM-YFP constructs. The concentration of the TNKS1 or TNKS2 SAM-CFP constructs was kept constant at 200 nM and mixed with increasing concentrations of SAM-YFP constructs of TNKS1 or TNKS2 and FRET fluorescence emissions (EmFRET) were determined. **(a)** TNKS1-SAM(E1050K)-CFP was mixed with TNKS1-SAM(Y1073)-YFP. The determined K_d is 560 nM. **(b)** TNKS1-SAM(E1050K)-CFP was mixed with TNKS2-SAM(Y920A)-YFP. The determined K_d is 300 nM **(c)** TNKS1-SAM(E1050K)-CFP was mixed with TNKS2-SAM(Y920A)-YFP. The determined K_d is 300 nM. Data shown are mean ± standard deviation with number of replicates n=4.

Buffer optimization

All buffer optimizations were carried out with 250 nM FRET donors and 500 nM FRET acceptors. To find a suitable buffer system for the assay, several conditions were tested. Per condition, 4 replicates were prepared in 384-well plates with a volume of 20 µl per well. First, we tested the effect of the pH on the assay signal (**Figure S4a, b**). We used Bis-Tris-Propane as buffer for this experiment due to its wide buffer capacity range. The pH values in a range of 6 to 9.5 were tested in increments of 0.5. The rFRET signals of the respective FRET pairs CFP-TNKS2 ARC4/YFP-TBM and TNKS2-SAM(E897K)-CFP/TNKS2-SAM(Y920A)-YFP at the different pH conditions were tested. Controls with YFP to test for unspecific effects on the FRET signals were prepared under the same conditions. For the TNKS2 ARC4-TBM FRET pair, the optimal rFRET signal was found at a pH of 6.5, while pH 6.0 and 7.0 showed signals only slightly below that. Starting from pH 6.5 the signals decreased with increasing pH, with pH 9.5 showing the lowest signal. Similarly, the TNKS2 SAM FRET pair showed its highest rFRET signal at pH of 6.0 and a decrease of rFRET signal strength was seen with an increase of pH values. As the assay will be used to find small molecules of the respective interactions for *in vivo* studies, we decided to continue testing of further buffer conditions at a neutral pH of 7.0, closely resembling the intracellular pH of the cytoplasm.

Next, different buffering agents were tested, as these can influence the activity and binding of biomolecules (**Figure S4c, d**). For the TNKS2 ARC4-TBM FRET pair, all buffering agents had similar signals. The TNKS2 SAM FRET pair showed the highest signal in Bis-Tris-Propane and imidazole. For consistency, we decided to use Bis-Tris-Propane for further buffer optimization with both assay systems.

To further optimize the assay conditions, common buffer additives were used to evaluate their effect on the signal strength of the assay systems. Each additive was tested in three concentrations. A control with YFP was tested as well for both assay systems. The rFRET signal of each condition is shown as a percentage relative to the signal of controls without additives (**Table S2**). For each assay system, the salts NaCl and $(NH_4)_2SO_4$ at concentrations of 250 mM to 750 mM drastically reduced the rFRET signal in all concentrations tested. Likely the binding of the FRET pairs and therefore the rFRET signal is inhibited by higher ionic strengths of the solution. In both tested systems, glycerol concentrations of 3% to 9% slightly reduced the rFRET signal by up to 16%. BSA did not show any effect on the signal of the TNKS2 ARC4-TBM FRET pair, however it did show an inhibiting effect on the TNKS2 SAM FRET pair. Several PEGs as additives were tested to serve as crowding agents and prevent binding of the proteins to tube- and plate materials. All tested PEGs showed an increase in the FRET signal. Finally, we decided to include 3%(w/v) PEG 20,000 in our assay buffer system. This amount enhances FRET signal and serves at the same time as agent blocking adhesion of biomolecules to plastics (Liu et al., 2013).

References:

Liu, B., Huang, P.-J.J., Zhang, X., Wang, F., Pautler, R., Ip, A.C., and Liu, J. (2013). Parts-per-Million of Polyethylene Glycol as a Non-Interfering Blocking Agent for Homogeneous Biosensor Development. Anal. Chem. *85*, 10045–10050.

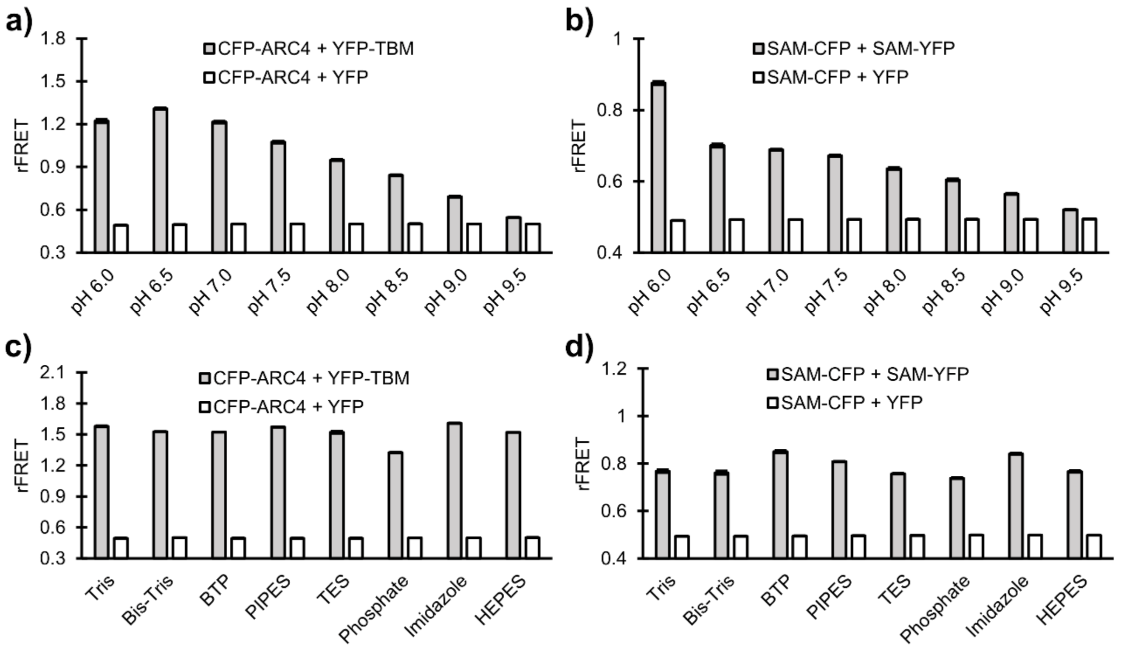

Figure S4: Buffer pH and buffering agent optimization. The pH-dependence of the assay signal was tested in Bis-Tris-Propane buffer with pH ranging from 6.0 to 9.5 with the TNKS2 ARC4-TBM FRET pair **(a)** and the TNKS2 SAM FRET pair **(b)**. Controls with YFP were included in both systems. The buffer systems Tris, Bis-Tris, Bis-Tris-Propane (BTP), PIPES, TES, sodium phosphate, imidazole and HEPES were tested at pH 7.0 with the TNKS2 ARC4-TBM FRET pair **(c)** and the TNKS2 SAM FRET pair **(d)**. Data shown are mean ± standard deviation with number of replicates n=4.

Table S2: Buffer additive optimization. Optimization was done with the FRET pairs TNKS2 ARC4-TBM and TNKS2 SAM. All additives were tested in 10 mM Bis-Tris-Propane (pH 7.0), 0.01% Triton-X100 and 0.5 mM TCEP. For each additive, the ratiometric FRET signal was measured and the signal was calculated relative to the controls containing YFP-TBM or SAM(E897K)-YFP (100% signal) and YFP (0% signal) in buffer without additives. Data shown are mean ± standard deviation (SD) with number of replicates n=4.

Buffer additive	Concentration	CFP-ARC4 (TNKS2) / YFP-TBM		CFP-ARC4 (TNKS2) / YFP		TNKS2-SAM-CFP / TNKS2-SAM-YFP		TNKS2-SAM-CFP / YFP	
		rFRET [%]	SD ±	rFRET [%]	SD ±	rFRET [%]	SD ±	rFRET [%]	SD ±
NaCl	250 mM	35.2	0.27	0.0	0.14	21.3	0.71	-0.29	0.67
	500 mM	23.6	0.23	0.23	0.24	11.2	0.74	-0.19	0.71
	750 mM	20.2	0.23	0.0	0.13	8.1	0.69	0.1	0.59
$(NH_4)_2SO_4$	250 mM	23.7	0.62	0.05	0.12	12.0	0.63	0.43	0.70
	500 mM	24.5	0.29	0.25	0.19	8.5	0.70	0.62	0.56
	750 mM	34.7	0.21	0.13	0.28	10.1	0.55	0.78	1.19
Glycerol	3%(v/v)	95.5	0.37	0.28	0.20	93.8	1.26	0.87	0.72
	6%(v/v)	89.7	0.60	0.30	0.14	90.8	1.21	0.24	0.62
	9%(v/v)	84.1	0.39	-0.1	0.15	90.3	0.67	0.2	0.62
BSA	0.5 mg/ml	101	0.53	0.11	0.12	82.8	1.14	0.29	0.67
	1.0 mg/ml	100	0.59	0.34	0.22	74.9	0.77	0.88	0.55
	1.5 mg/ml	100	0.90	0.0	0.16	66.2	0.65	0.1	0.59
PEG3350	3%(v/v)	116	0.93	0.49	0.22	129	0.75	1.3	0.64
	6%(v/v)	129	0.67	1.4	0.21	167	1.16	2.4	0.93
	9%(v/v)	144	2.0	1.8	0.18	219	0.9	5.9	0.60
PEG6000	3%(v/v)	118	0.86	0.46	0.20	135	4.12	0.86	0.56
	6%(v/v)	134	0.46	1.4	0.31	180	1.18	2.9	0.67
	9%(v/v)	143	0.88	1.7	0.16	271	1.80	8.2	0.70
PEG8000	3%(v/v)	119	0.37	0.39	0.28	136	0.87	1.2	0.76
	6%(v/v)	134	0.38	1.5	0.19	186	1.80	2.6	0.78
	9%(v/v)	147	1.1	2.2	0.35	296	1.2	8.2	0.89
PEG20,000	3%(v/v)	119	0.51	0.44	0.13	137	0.63	0.91	0.65
	6%(v/v)	132	0.72	1.6	0.16	188	1.16	2.4	0.58
	9%(v/v)	144	1.4	1.9	0.21	299	2.5	9.0	0.74

Effect of GdnHCl on CFP and YFP fluorescence

In this study, 1 M GdnHCl was used as a control to disrupt the protein-protein interactions, thereby leading to a loss of the ratiometric FRET signal. To ensure that these conditions have little effect on the fluorescent proteins CFP and YFP, we measured their fluorescence intensities (**Figure S5a, b**) and emission spectra (**Figure S5c, d**) at different concentrations of GdnHCl. Even at a concentration of 6 M GdnHCl, the fluorescence intensities were reduced by only about 25% compared to the control containing no GdnHCl. Lower concentrations had little or no observed effect on the fluorescence of CFP or YFP, respectively.

Fluorescence intensities of 1 µM CFP or YFP were measured in 384-well plates in replicates n=4 and 20 µl per well. For CFP, an excitation wavelength of 430 nm (5 nm bandwidth) and an emission wavelength of 477 nm (5 nm bandwidth) were used. For YFP, an excitation wavelength of 477 nm (5 nm bandwidth) and an emission wavelength of 527 nm (5 nm bandwidth) were used. Fluorescence spectra of 1 µM CFP or YFP were measured in black 96-well plates (Greiner) and 100 µl per well using the excitation wavelengths from above. Experiments were performed in assay buffer (10 mM Bis-Tris-Propane pH 7.0, 3%(w/v) PEG20,000, 0.01%(v/v) Triton-X100 and 0.5 mM TCEP) containing different concentrations of GdnHCl. Before the measurement, all samples were incubated for 1 h at room temperature.

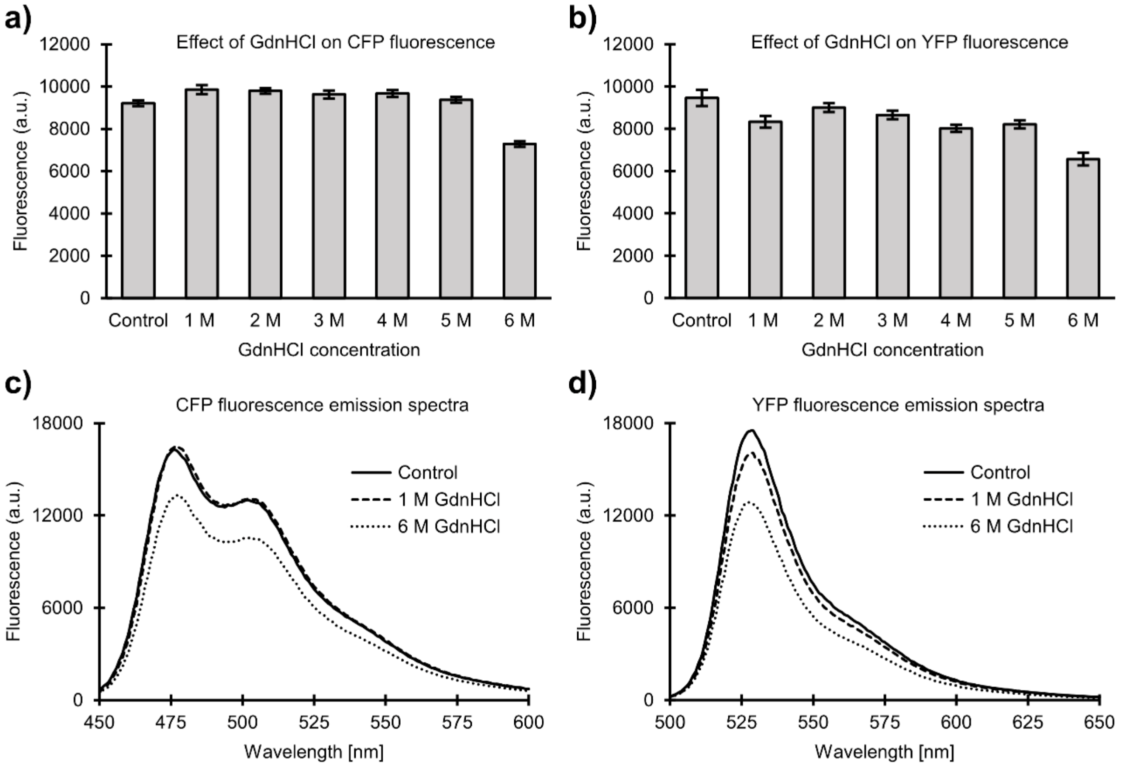

Supplementary Figure 5: Effect of GdnHCl on CFP and YFP fluorescence. The fluorescence emission intensities of (**a**) 1 µM CFP at 430 nm excitation and 477 nm emission and (**b**) 1 µM YFP at 477 nm excitation and 527 nm emission were measured at different guanidine hydrochloride (GdnHCl) concentrations. Data shown are mean ± standard deviation with number of replicates n=4. Fluorescence emission spectra are shown for (**c**) 1 µM CFP upon excitation at 430 nm and (**d**) 1 µM YFP upon excitation at 477 nm in 1 M GdnHCl (dashed line) and 6 M GdnHCl (dotted line). Additionally, controls containing no GdnHCl were measured. All samples were incubated for 1 h at room temperature before measurement.

Effect of DMSO on the assay systems

In many instances DMSO interferes with biochemical and cell-based assays. Since compounds in screening libraries are commonly dissolved in DMSO, we tested the effect of DMSO on the assays. The proteins were prepared in the optimized assay conditions containing a range of different DMSO concentrations up to 5% (**Figure S6**). For the TNKS2 ARC4-TBM FRET pair, the ratiometric FRET signal was reduced in conditions containing ≥1% DMSO, however even at 5% DMSO the signal decreased only 25% compared to the condition without DMSO. For the TNKS2 SAM FRET pair, even DMSO concentrations up to 5% did not affect the signal drastically. For the assays described here, DMSO concentrations in all conditions and controls should always be the same to exclude effects of DMSO. Per condition, 4 replicates were prepared in 384-well plates with a volume of 20 µl per well.

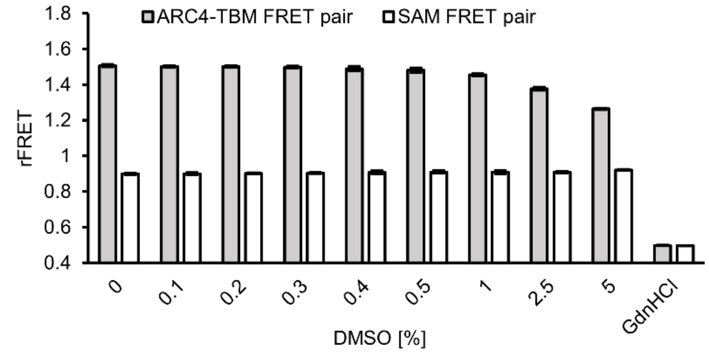

Figure S6: Effect of different DMSO concentrations on the assay systems. DMSO in different concentrations (0, 0.1, 0.2, 0.3, 0.4, 0.5, 1, 2.5 and 5%) was added to the TNKS2 ARC4-TBM FRET pair or TNKS2 SAM FRET pair systems and incubated for 1 h. Additionally, a control containing 1 M GdnHCl was included to show the loss of FRET signal. Data shown are mean ± standard deviation with number of replicates n=4.

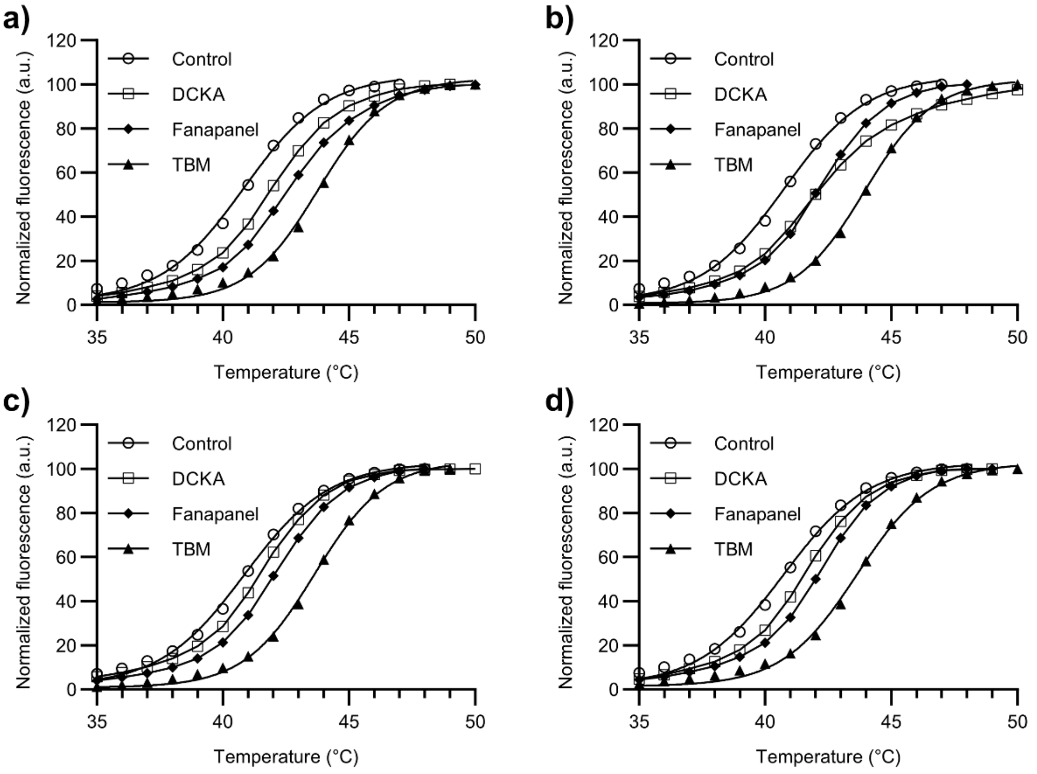

Figure S7: Four replicate measurements with differential scanning fluorimetry for hit compounds of TNKS2 ARC4. Unlabelled TNKS2 ARC4 was mixed with 100 µM 5,7-dichlorokynurenic acid (DCKA), Fanapanel and REAGDGEE TNKS binding motif peptide (TBM). Additionally, controls without compounds were prepared. All conditions contained 1%(v/v) DMSO. Four replicates per condition were prepared. The melting temperatures (T_m) of ARC4 were calculated: **(a)** Control: T_m = 40.83 °C, DCKA: T_m = 41.82 °C, Fanapanel: T_m = 42.51 °C, TBM: T_m = 43.76 °C. **(b)** Control: T_m = 40.75 °C, DCKA: T_m = 42.05 °C, Fanapanel: T_m = 42.06 °C, TBM: T_m = 43.93 °C. **(c)** Control: T_m = 40.86 °C, DCKA: T_m = 41.34 °C, Fanapanel: T_m = 41.98 °C, TBM: T_m = 43.59 °C. **(d)** Control: T_m = 40.74 °C, DCKA: T_m = 41.46 °C, Fanapanel: T_m = 42.03 °C, TBM: T_m = 43.64 °C.

Estimation of binding affinities of hit compounds to ARC4

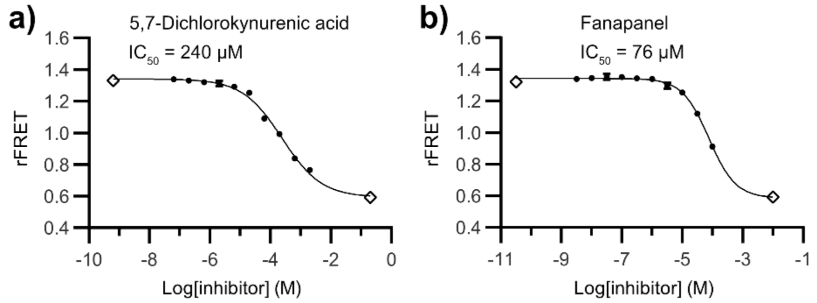

Figure S8: IC_{50} measurements of two hit compounds with ARC4-TBM FRET pair. The 50 nM of CFP-fused TNKS2 ARC4 and 100 nM YFP-TBM and mixed with increasing concentrations of 5,7-dichlorokynurenic acid **(a)** and Fanapanel **(b)**. Controls were placed 2-logarithm units above or below the highest or lowest compound concentrations (open diamonds). Data shown are mean ± standard deviation with number of replicates n=4.

The binding affinities of the compounds were estimated from obtained IC_{50} (**Figure S8**), using the Cheng-Prusoff equation:

$K_i = IC_{50} / (1 + ([L]/K_d))$

Where:

IC_{50} Half-maximal inhibition when compound dilutions are mixed with ligand-receptor pair (YFP-TBM/CFP-ARC4); 240 µM (5,7-dichlorokynurenic acid) or 76 µM (Fanapanel)

[L] Concentration of ligand (YFP-TBM, 100 nM) used in IC_{50} experiment

K_d Dissociation constant of ligand (YFP-YBM) to receptor (CFP-ARC4) (35 nM, estimation from FRET-based binding affinity studies, **Figure S2**)

Thus, we estimated the K_i values of 5,7-dichlorokynurenic acid and Fanapanel for TNKS2 ARC4 to be 62 µM and 20 µM, respectively.

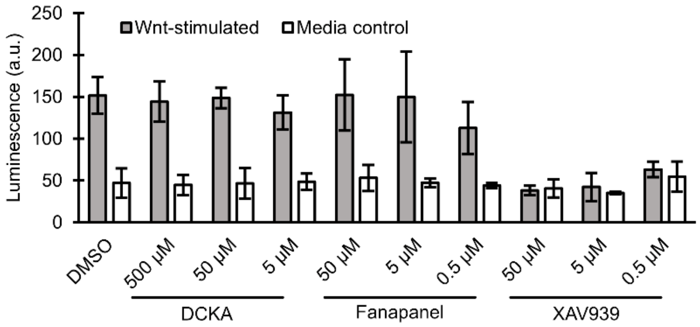

Figure S9: TCF/LEF Wnt-reporter assay. The TCF/LEF HEK293 cells were stimulated with Wnt-conditioned or control media in presence of compounds 5,7-dichlorokynurenic acid (DCKA), Fanapanel and a known potent TNKS inhibitor XAV939. All conditions contained 0.5 % DMSO. Higher luminescence values correspond with a higher Wnt-signalling activity. Data shown are mean ± standard deviation with number of replicates n=3.
